# Workplace violence against nurses in the Gambia: mixed methods design

**DOI:** 10.1186/s12913-017-2258-4

**Published:** 2017-04-28

**Authors:** Ebrima J. Sisawo, Saide Yacine Y. Arsène Ouédraogo, Song-Lih Huang

**Affiliations:** 1 The University of the Gambia, Brikama, The Gambia; 2 International Health Program, National Yang-Ming University, Taipei, Taiwan; 3 Institute of Public Health, National Yang-Ming University, No.155, Sec.2, Li-Nong Street, Taipei, 112 Taiwan, Republic of China

**Keywords:** Workplace violence, Secondary health care facilities, Nurses, Gambia

## Abstract

**Background:**

The aim of this study was to assess the prevalence, perpetrators and factors associated with workplace violence against nurses in public secondary health care facilities from two health regions in the Gambia.

**Methods:**

Data was collected from 219 nurses using self-administered questionnaire and 35 face-to-face interviews. The data collection was conducted between July and September 2014 in 14 public secondary health care facilities.

**Results:**

A sizable majority of respondents (62.1%) reported exposure to violence in the 12 months prior to the survey; exposure to verbal abuse, physical violence, and sexual harassment was 59.8%, 17.2%, and 10% respectively. The perpetrators were mostly patients’ escorts/relatives followed by patients themselves. Perceived reasons of workplace violence were mainly attributed to nurse-client disagreement, understaffing, shortage of drugs and supplies, security vacuum, and lack of management attention to workplace violence.

**Conclusions:**

Nurses in the Gambia are at a relatively high risk of violent incidents at work. Policies and strategies that are sensitive to local circumstances and needs should be developed for the prevention of workplace violence.

**Electronic supplementary material:**

The online version of this article (doi:10.1186/s12913-017-2258-4) contains supplementary material, which is available to authorized users.

## Background

Workplace violence is defined as incidents where staff are abused, threatened or assaulted in circumstances related to their work, including commuting to and from work, involving an explicit or implicit challenge to their safety, well-being or health [1]. Studies have documented workplace violence as one of the most complex and dangerous occupational hazards facing nurses. The International Labor Office (ILO)/International Council of Nurses (ICN)/World Health Organization (WHO)/Public Services International (PSI) joint program on workplace violence in the health sector in 2003 indicated that nurses are 3 times more likely, on average, to experience violence in the workplace than other occupational groups [1]. Nurses are subjected to verbal and physical abuse so frequently that these events are often accepted as “part of the job” [2].

An Egyptian study indicated that 69.5% and 9.3% of nurses faced verbal and physical violence, respectively [3]. Pai and Lee revealed that the main forms of work-related-violence reported by nurses in Taiwan were physical violence, verbal abuse, bullying/mobbing and sexual harassment [4]. Most studies on this subject highlighted patients’ relatives/escorts and patients themselves as perpetrators of violence [4–7]. Increased number of evidence indicates that violence is now considered to be a major occupational hazard for nurses worldwide [8]. The problem of violence in health care is not new; it has probably always been a part of nursing [9]. The joint ILO/ICN/WHO/PSI research findings indicate that in developing and transition countries, more than half of the health sector personnel experienced at least one incident of physical or psychological violence. The worry, fear and anxiety of being exposed to verbal and physical violence are common among nurses [10].

This study focuses on nurses working in public secondary health care facilities in the Gambia. Gambian nurses fall under four main categories: State Registered Nurse (SRN), State Enrolled Nurse (SEN), Community Health Nurse (CHN) and Community Nurse Attendant (CNA) [11]. SRNs are the first level of general nurses who performed general patient care. They also handle management and supervision roles in public secondary health care facilities. The SENs are the second level of general nurses whose training is not as rigorous as that of the SRNs. They assist the SRNs in carrying out basic nursing care procedures for patients. This allows the SRNs to concentrate on more complex nursing care procedures. The CHNs live and work for the most part in primary health care villages, supervising the village health workers and traditional birth attendants. Some of the CHNs are posted to the reproductive and child health teams in public secondary health care facilities. The CNAs have basic nursing skills with no formal training in nursing decision-making. They receive on-the-job training to become nurse assistants. An 18 month advanced midwifery program is available to SRNs, SENs and CHNs to become midwives. Human Resources for Health report in 2012 indicate a total of 1090 nurses working in the public health sector in the Gambia. Of these, 549 nurses were distributed in primary, secondary and tertiary levels of care in Western I and Western II Health Regions [12]. The proportion of nurses in the Gambia is 3.2/10,000 population [12] which is below the WHO’s minimum threshold of 23 doctors, nurses and midwives per 10,000 population. There has been a high attrition within the health sector especially skilled staff such as nurses, doctors, public health officers and midwives both internally and externally and was estimated between 30% and 50% per two years [11]. A study conducted in 2002 linked attrition of nurses to poor remuneration and incentives, lack of housing facilities, poor working environment and conditions including equipment, supplies and logistics, lack of/inability to develop in one’s career and heavy workload with same remuneration. Another study in 2016 reported 87% prevalence of perceived effort-reward imbalance among health care professionals in the Gambia including nurses [13]. This study indicates that nurses reported higher perceived efforts, gain fewer rewards and express a higher degree of over commitment at work than environmental health officers. Low job satisfaction and high attrition of nurses could exert work pressure on nurses due to low staffing-patient ratio consequently affecting their service behaviour.

Health services in the Gambia consists of three levels [11]. The primary level is the first point of contact with the health system at community level. It provides mainly preventive care and treatment of minor ailments. The primary level consists of health care villages that have been selected from those with a population of 400 or occasionally from ones located in relatively isolated areas [11]. Health care delivery at primary level is provided by Village Health Workers (VHWs) and Traditional Birth Attendants who are both supervised by community health nurses (CHNs). Functioning in parallel with the primary health care villages are the basic health facilities/secondary health care facilities (the focus of the current study). The secondary level includes major and minor health centers that provide preventive (including reproductive and child health services, curative and inpatient services). Major health centers provide basic surgery if they have the staff to do so but minor health facilities do not do surgery (except eye surgery, which is handled by the National Eye Care Program trained staff). Nurses constitute the majority of staff composition in public secondary health care facilities. There are positions for pharmacy assistant, laboratory assistant, environmental health officers and other support staff including orderlies and ambulance drivers. The tertiary level comprise of hospitals which provide all services including specialist care and/or services. The hospitals provide care for patients whose conditions cannot be handled at the basic/secondary health care facilities. The staffing profile of hospitals (tertiary level) consists of all categories of nurses, doctors (including specialists), X-ray, pharmacy, laboratory, medical record staff and orderlies. A few dozen private health clinics and many pharmacies also diagnose and prescribe treatment, particularly in the urban area. These are not integrated into the government system, and provide services for fees paid by the patients.

The current study focuses on nurses because they represent approximately 75% of the total healthcare workforce in the Gambia [11]. They are the most available personnel throughout health care settings in the country making them invaluable human resources for the health care sector. They serve as front-line health care providers which increases their contact with the public. Consequently, this increases their risk of exposure to violent behaviors from aggressive patients and the public. Although nurses worldwide experienced violence in their workplace, how nurses experience violence in the Gambia remains unclear. To the best of the authors’ knowledge, there is no information on occupational violence towards Gambian nurses in published literatures. There is no incident reporting procedure or policy to monitor workplace violence in the Gambian nursing workforce. The absence of such policies renders it difficult to document the extent of the problem.

Given that this research represents the first study to explore workplace violence against nurses in the Gambia, the findings could serve as baseline information for further large-scale studies to closely examine the problem of violence in the Gambian nursing workforce. The data generated could be valuable for health care managers and policy makers in planning violence reduction interventions. By extension, the findings could contribute to existing literature in the developing world where the subject is under researched. Given the aforementioned gaps and concerns, this study was conducted to address the following aims: First, to determine the prevalence and perpetrators of workplace violence against nurses in the Gambia. Second, it attempts to identify possible factors associated with workplace violence against nurses in the Gambia.

## Methods

### Study design and selection of participants

This study employed quantitative and qualitative designs to address the study aims. The study was conducted in two (2) out of the seven (7) health administrative regions in the Gambia. The regions in question were Western I and Western II Health Regions. The two regions account for 52% (549 nurses) of the total nurse population working in public health care facilities in the Gambia [12]. Public health care facilities include hospitals (tertiary level) major and minor health facilities (secondary level) and primary health care villages (primary level). This study was focused on secondary health care facilities (major and minor health facilities). These facilities provide outpatient (including reproductive and child health services) and inpatient services (but not specialist care). The study targeted all 298 nurses that were working in the fourteen (14) public secondary health care facilities in Western I and Western II health regions. All nurses with at least one (1) year of work experience and who were present on the day of the survey were included, while students and nurse trainees were excluded. Overall, 223 out of the 298 nurses met our inclusion criteria; informed consent was obtained from 221 nurses and there were 2 incomplete questionnaires. This report is based on data from the remaining 219 nurses corresponding to a response rate of 98.2%.

For the interviews, purposive sampling was used to select respondents. In each health facility three (3) nurses were invited to participate in a face-to-face interview. Participants were selected on the basis of either experiencing or witnessing workplace violence 12 months prior to the study. Forty-two (42) nurses were approached but seven (7) declined participation on personal grounds. Consequently, analysis in this study was based on 35 nurses who consent participation. Permission to conduct this study was obtained from the Gambia Government/Medical Research Council Joint Ethics Committee and the Research and Publication Committee of the University of the Gambia (RePUBLIC).

### Survey instrument

Data collection took place between July and September 2014. For the quantitative part, data was collected using a pretested and self-administered questionnaire that was adapted from the International Labor Office (ILO), World Health Organization (WHO), International Council of Nurses (ICN) and Public Services International (PSI) joint program on workplace violence in the health sector [1]. The tool was modified to suit the study objectives and cultural context of the Gambia. The tool was maintained in its English version since Gambian nurses are able to read and write in English. An expert in Occupational Safety at the National Yang-Ming University in Taiwan and another at the University of the Gambia examined the content validity and reliability of the tool. They were asked to assess the questionnaire for its clarity, relevance, comprehensiveness, and sensitivity to Gambian culture. Pretesting of the tool was conducted with 20 nurses who were subsequently excluded from the study. Workplace violence was regarded when the study participants experienced at least one type of violence such as physical violence, verbal abuse, or sexual harassment in circumstances related to their work twelve (12) months prior to the study. Physical abuse was defined as being hit, pushed, beaten, kicked, slapped, stabbed, shot, bitten and/or pinched in the workplace. Verbal abuse was regarded as being shouted at, insulted, intimidated, embarrassed, blamed or verbally disrespected in the workplace. Sexual harassment was defined as being stared at, whistled at, embraced, kissed, touched inappropriately, unwanted request for sexual favors/dates, unwelcome verbal sex-based jokes/comments, invited to date with promise of promotion/other privileges or sexually attacked. The questionnaire was used to obtain the following information: personal and workplace data of respondents, exposure to workplace violence, perpetrator, location and time of violence. A face-to-face interview was conducted by the lead author with thirty-five (35) nurses to gather information on the circumstances of the violence and perceived factors associated with the occurrence of violence. The interviews were guided by these grand tour questions: 1. Describe one incident of workplace violence you experienced within the last 12 months 2. What do you think was the reason why the incident happened? What do you think could be done to prevent such incidents from happening?

### Statistical analysis

Data from the questionnaire was entered into Epi info and analysed with SPSS version 21. Descriptive statistics was used to analyse the socio-demographic and professional characteristics of the respondents, as well as the prevalence and perpetrators of workplace violence. Crude odds ratios & 95% confidence intervals was used to assess potential associations between exposure to physical violence, verbal abuse and sexual harassment and respondents’ characteristics including age, gender, marital status, region, area (rural/urban) occupational/nurse cadre, and number of co-workers. Adjustment was made for the same pre-mentioned covariates using a logistic regression model; the dependent variables being exposure to physical violence, verbal abuse and sexual harassments. A *p* value <0.05 was considered statistically significant in the analysis.

The interviews were transcribed verbatim using *Transcribe* software. At first, the transcripts were coded by highlighting significant statements. Through team discussions, codes with the same focus were combined into themes. Subsequently 5 themes were developed, which highlighted factors associated with workplace violence against nurses. In this report, the participants’ names and their health facilities are pseudonyms to preserve anonymity.

## Results

A total of 219 out of the 223 questionnaires sent were returned (response rate = 98.2%) from 29 State Registered Nurses, 52 State Enrolled Nurses, 64 Community Health Nurses and 74 Community Nurse Attendants. The profile of the nurses included in this study is provided in Table 1. A sizable majority (68%) of the respondents came from Western I Health Region. Most of the respondents were females (73.1%), older than 30 years (63.1%) and married (82.2%). Nearly 66% of the participants are professional nurses (received certified training in nursing [Midwives and General Nurses]), with the experience of more than five years (69%) in nursing. Besides, two-third (66.7%) of them indicated to have five or less colleagues as regular co-workers in their units.

**Table 1:** Socio-demographic and professional characteristics of respondents (*N* = 219)

Characteristic	N	%
Region		
Western I	149	68
Western II	70	32
Gender		
Male	59	26.9
Female	160	73.1
Age groups		
≤ 30 years	78	35.6
31–40 years	84	38.4
≥ 41 years	54	24.7
Missing	3	1.3
Marital status		
Single	39	17.8
Married	180	82.2
Occupational cadre		
^a^Midwives	72	32.9
^b^General nurse	73	33.3
Nurse Attendant	74	33.8
Length of service		
1–5 years	66	30.1
6–10 years	42	19.2
11–15 years	38	17.4
Above 16 years	71	32.4
Missing	2	0.9
Number of co-workers		
≤ 5	146	66.7
6–10	44	20.1
≥ 11	29	13.2

^a^SRNs, SENs and CHNs with Midwifery qualification

^b^SRNs, SENs and CHNs without Midwifery qualification

The prevalence of workplace violence is presented in Table 2. Overall, 62.1% of the respondents reported exposure to workplace violence in the 12 months prior to the survey. Of them, 17.4% reported exposure to physical violence, nearly 60% reported verbal abuse while 10% reported to have been sexually harassed. Findings further revealed that 22.5% of the victims of physical violence had encountered 2–4 episodes of physical violence and almost 23% of them had been threatened with a weapon. Moreover, 46% of the respondents indicated having witnessed physical violence directed towards peers.

**Table 2:** Prevalence of violence reported by respondents in the previous 12 months (*N* = 219)

Exposure to violence	Physical violence		Verbal abuse		Sexual harassment		Overall prevalence	
	N	%	n	%	n	%	n	%
Yes	38	17.4	131	59.8	22	10	136	62.1
No	181	82.6	88	40.2	197	90	83	37.9

Nurses who had experienced workplace violence were asked to indicate the source of violence (Table 3). The respondents described perpetrators of verbal abuse as mainly patients’ relatives/escorts followed by patients. Similarly, the most frequent source of physical violence was patients’ relatives/escorts and patients. Some nurses identified other staff members as perpetrators of verbal abuse and physical violence. For sexual harassment, patients’ relatives/escorts remained the primary aggressors, followed by the general public and patients.

**Table 3:** Perpetrator, location and time of the most recent incidents experienced by nurses (*N* = 219)

	Verbal abuse	Physical violence	Sexual harassment
	*n* (%)	*n* (%)	*n* (%)
Exposure to violence (Yes)	131(59.8)	38 (17.4)	22 (10)
Perpetrator			
Patient	44 (33.6)	15 (39.5)	4 (18.2)
Patients’ relative/escort	71 (54.2)	18 (47.4)	9 (40.9)
Staff member	7 (5.34)	3 (7.9)	2 (9.1)
Management/supervisor	4 (3.05)	1 (2.6)	1 (4.5)
Colleague from other health facility	1 (0.76)	1 (2.6)	0 (0.0)
General public	4 (3.05)	0 (0.0)	6 (27.3)
Location of incident			
Outpatient department	78 (59.5)	18 (47.4)	11 (50.0)
Admission ward	23 (17.6)	11 (28.9)	4 (18.2)
Maternity ward	21 (16.0)	8 (21.1)	1 (4.5)
Elsewhere	9 (6.9)	1 (2.6)	6 (27.3)
Time of incident			
Morning shift (8 am–2 pm)	81 (61.8)	26 (68.4)	8 (36.4)
Afternoon shift (2 pm–8 pm)	25 (19.1)	6 (15.8)	7 (31.8)
Night shift (8 pm–8 am)	23 (17.6)	5 (13.2)	7 (31.8)
Missing	2 (1.5)	1 (2.6)	0 (0.0)

When asked about where the incident of verbal abuse took place, approximately 60% of victims indicated the outpatient department, whereas 17.6% cited the admission ward and 16% indicated the maternity ward. Similarly, the most frequent location for physical violence was the outpatient department followed by the admission ward and the maternity ward. The majority of the incidents of sexual harassment also occurred in the outpatient department with ‘on the way to work’ ranking second. Most violence incidents took place in the morning shift, followed by the afternoon shift.

Table 4 shows the results of the unadjusted and multivariate-adjusted risk estimates for exposure to physical violence, verbal abuse and sexual harassment. Unadjusted analysis indicates that exposure to physical violence was significantly associated with respondents who were nurse attendants (OR = 2.6; 95% CI = 1.04-6.37) and those working in Western I Health Region (OR = 2.9; 95% CI = 1.16–7.35). On verbal abuse, nurses who were single had 60% less odds of reporting incidents as compared to married nurses (OR = 0.4; 95% CI = 0.22-0.90). Also, General nurses had higher odds of reporting sexual harassment than midwives (OR = 4.1; 95% CI = 1.09–15.30). In multivariate analysis, only three characteristics remained significantly associated with exposure to workplace violence. In particular, respondents who were working in Western I Region were almost 3 times more likely to report physical violence than those in Western II Region (OR =2.8; 95% CI = 1.09–7.20). Respondents who were single were less likely to report verbal abuse than married nurses (OR = 0.3; 95% CI = 0.12–0.62). Nurses with five or less co-workers had significantly higher odds of reporting verbal abuse than those with 11 co-workers and more (OR = 3.0; 95% CI = 1.22–7.38).

**Table 4:** Un-adjusted and multivariate adjusted odds ratios for exposure to violence among respondents in the past 12 months

Variable	Physical violence		Verbal abuse		Sexual harassment	
	Unadjusted OR (95% CI)	Adjusted OR (95 CI)	Unadjusted	Adjusted	Unadjusted	Adjusted
			OR (95% CI)	OR (95% CI)	OR (95% CI)	OR (95% CI)
Area						
Urban	3.3 (0.43, 26.11)	4.3 (0.52, 34.72)	1.5 (0.56, 4.26)	1.9 (0.66, 5.47)	1.7 (0.22, 13.77)	1.7 (0.21, 13.92)
Rural	1	1	1	1	1	1
Gender						
Male	1.5 (0.72–3.23)	1.5 (0.68–3.32)	1.6 (0.85–2.99)	1.9 (0.95–3.64)	1.3 (0.50–3.37)	1.2 (0.43–3.30)
Female	1	1	1		1	1
Age group						
≤ 30 years	1.4 (0.59–3.76)	1.3 (0.47–3.64)	1.5 (0.74–2.99)	2.0 (0.89–4.65)	2.9 (0.61–14.58)	2.6 (0.49–13.92)
31–40 years	0.9 (0.36–2.52)	1.2 (0.45–3.40)	1.5 (0.76–3.01)	1.7 (0.78–3.52)	3.5 (0.74–16.70)	3.6 (0.73–17.56)
≥ 41 years	1	1	1	1	1	1
Marital status						
Single	1.3 (0.54–3.08)	0.9 (0.37–2.49)	0.4 (0.22–0.90)*	0.3 (0.12–0.62)*	1.0 (0.33–3.23)	0.9 (0.26–3.18)
Married	1	1	1	1	1	1
Occupational Cadre						
Nurse attendant	2.6 (1.04–6.37)*	1.9 (0.71–5.07)	0.8 (0.41–1.53)	0.6 (0.30–1.31)	2.8 (0.71–10.96)	2.1 (0.48–9.21)
General nurse	1.6 (0.60–4.11)	1.2 (0.42–3.67)	1.6 (0.79–3.07)	1.2 (0.53–2.56)	4.1 (1.09–15.30)*	4.1 (0.98–17.38)
Midwives	1	1	1		1	1
Region						
Western I	2.9 (1.16–7.35)*	2.8 (1.09–7.20)*	1.3 (0.72–2.28)	1.6 (0.89–3.06)	1.0 (0.39–2.59)	0.9 (0.32–2.32)
Western II	1	1	1	1	1	1
Number of co-workers						
≤ 5	1.1 (0.40–3.25)	1.5 (0.46–4.89)	2.1 (0.95–4.75)	3.0 (1.22–7.38)*	0.9 (0.23–3.18)	0.9 (0.19–4.95)
6–10	0.6 (0.16–2.35)	1.1 (0.25–4.95)	0.9 (0.35–2.29)	1.2 (0.42–3.36)	1.4 (0.31–5.97)	2.1 (0.35–12.56)
≥ 11	1		1	1	1	1

*Statistically significant (*p* value <0.05)

### Factors associated with workplace violence

Interviews were conducted with 35 nurses regarding the circumstances of the violence incidents they experienced or witnessed. From their descriptions, five common themes were identified which pointed out factors associated with workplace violence. In presenting the results, quotes were used to reflect participants’ voices.

### Nurse-client disagreement

Responses from participants indicate lack of cooperation from patients and their escorts as a precursor to violent incidents. In some instances, patients or their escorts refused nurses’ instructions culminating in abuses directed towards nurses. This was reflected in Foday’s response as to how he received abusive utterances from a patient escort*. “We were conducting ward round and there were many escorts in the labor ward at the time. We asked them to go out and there was this lady [a patient escort] who refused to comply. She was furious with our directive and started telling me you don’t know yourself; you are stupid”.* (Foday/male Registered Nurse Midwife, Facility WII-1).

Divergence in nurses’ and escorts’ interpretation of an emergency case provoke both verbal and attempted-physical abuse towards participants. There were instances when escorts demanded their patients to be accorded emergency care and had such requests turned down by nurses. Nurses’ reports indicate that some escorts would present their patients as emergency cases on the pretext of bypassing long queues and waiting time for care. Fanta, a female Officer-in-Charge from facility WI-8, frustratingly recounted how she got insulted by a patient company. She reflected an encounter in which she was on duty alone and attending to a baby with severe birth asphyxia. While resuscitating the baby, a man accompanying an injured patient arrived and demanded immediate attention for his patient. Fanta’s attempt to make the man understand that his injured patient was not an emergency case irritated the escort. *“His patient sustained laceration which was not deep and not bleeding either. So I asked the escort to allow me some time to revive the baby but this does not go down well with him. He insisted I should turn my attention to his patient. When I did not, he started telling me all sorts of nonsense, all sorts of bad words you could imagine”.*

### Nurses’ workplace manners

In some cases, participants labelled nurses as the initiators of workplace violence. One of the commonly cited reasons for this was nurses uttering indecent languages towards clients. Musa, a Community Health Nurse from facility WI-7 indicated that sometimes, nurses give patients the cause to abuse them. He cited an encounter of a peer nurse who was slapped by a lactating mother for uttering obscene language to her. In describing the incident, he said: *“This woman was in labor. While screaming and yelling for help, the attending nurse was telling her ‘I was not there when you had this nice time [conjugal happiness] with your husband’. One week later, when the mother returns to the clinic for her child’s first immunization, she went into the labor ward to look for the nurse and gave her a very hot slap in retaliation”.*

Another eyewitness account was given by Ndey, a Registered Nurse Midwife from facility WI-2. She narrated the experience of a colleague: *“One of my colleagues who is a CHN General was attending to infants at the infant welfare clinic where infants are screened and treated for minor ailments. While attending to a patient, a waiting mother was asking the nurse several questions but got ignored by the nurse. This makes the mother so furious that she could not suppress her emotions and ended up grabbing the nurse’s neck. It was a real fight. We had to intervene to calm the situation”.*

In some instances, the attitude of nurses towards work provoked reactions from other staff members. The narration of Binta, a female Community Nurse Attendant at WII-5 describes how she was humiliated by her Officer-in-Charge (OIC) for reporting late to work. In her words *“There was a day I reported to work late. On arrival, before saying good morning, my Officer-In-Charge started to humiliate me. On that day I felt sad. I couldn't do anything. I couldn't talk to anyone because I felt so sad on that day. It affected my work on that day. I was not comfortable and felt disturbed on that day”*.

### Shortage of drugs and staff

Participants bemoaned drug shortage as a fundamental factor triggering aggressive reactions from clients. A significant fraction of prescribers in public secondary health care facilities in the Gambia are nurses. According to participants’ accounts, nurses often carry the blame when prescribed drugs are in short. Patients’ reactions to drug shortage took the form of abusive utterances and request to have their consultation fee returned. Sulayman, a male OIC at Facility WI-3 described a bitter confrontation his nurses had with a patient escort due to a prescribed drug that was not available. An OIC at facility WI-2 similarly stressed drug shortage as an important factor precipitating violent incidents towards nurses in his health center. He said: “*Well, there are so many violent acts in our work setting. But just to single out one very important factor. Sometimes, I do run the outpatient department. Sometimes you can prescribe a drug that is not available in the health center. As a result, they (patients) will insult you. They will say very nasty words. And it is not our duty to provide drugs but what can you do? Nothing. You just have to take it in good faith”.* (Biran, male OIC, facility WI-2).

Staff shortage stands out as a cardinal factor for long waiting time rendering clients in becoming bored and impatient. Such frustrations engender remarks from patients like *“nurses are inefficient and incompetent”* and in certain instances physical confrontation. In describing how a colleague was harassed by a patient, Lalia, a female Enrolled Midwife at facility WI-5 said: *“The patient visited the outpatient department and found the nurse busy—attending to someone who was unconscious. And that person wanted the nurse to leave the unconscious patient and attend to him. It was in the afternoon and the nurse was alone at the outpatient department. So when the nurse asked him to wait to finish attending to the unconscious patient since his condition was not an emergency, the boy [patient] started insulting the nurse. If there were enough nurses in that facility, that incident wouldn't have occurred”,* Lalia concluded.

In expressing how staff shortage makes nurses victims of abuse, a female CHN said: *“I was on night duty running both the labor ward and the outpatient department. This patient arrived and I was alone at the time. So the patient was furious for not attending to him immediately because at the time of his arrival, I had a labor case and I cannot leave that case because two lives were involved— the lady and her child. And this patient walked by himself to the health center and it was only headache and fever he had. So I explained my engagement to him but he couldn’t understand and started shouting, insulting but I ignored him and conducted my delivery and then attended to him afterwards. That's a common thing we often experience here. Even yesterday there was another one. For that one the patient decided to leave unattended*”. (Maimuna, female CHN General, facility WI-3).

### Lack of Security

Security gap was a commonly cited issue making health care facilities violent-prone. Most participants expressed inadequate or complete absence of security guards in their health centers. Those appointed to take charge of health center security were mostly the elderly with no professional training in security service. Lisa, a female Registered Nurse from facility WI-8 indicated that nurses in her health facility will feel safer if there are security guards. In her words: *“I think here we don't have security. I think if we have security and in case of any attempt to abuse a staff, we can call the security to intervene”.* Respondents’ feedbacks further indicated that the security vacuum creates a challenge in restricting patient visitors entering admission wards during restricted hours. When nurses attempt to prevent entry of patient visitors in the admission wards outside official visiting hours, they consequently received abusive utterances.

One of the participants, who was working in a private clinic (Family Planning Clinic) and then switched to the public sector, gave a comparison between the security situation at the private and public health facilities. She said *“Working in the private and the public sector are different. In the private sector, we are secured but for the public sector, nurses are not secured at all. When I came here* [her new workplace], *I have been hearing nurses complaining about people coming here, insulting them, and I witnessed one myself at Facility WII-1. People come, they fight them (nurses), they beat them, they insult them and after all nothing comes out of it. This doesn't happen in private health facilities”.* (Ndey, SEN Midwife WII-2).

### Policy vacuum and inadequate management support

The absence of a workplace violence policy both at the national and health facility levels affects nurses’ ability to prevent and respond appropriately to incidents of workplace aggressions. Feedbacks from participants suggest that some of their peers’ inability to communicate, understand and respond appropriately to clients’ needs often triggers aggressions from care seekers. And this was partly attributed to the lack of a well-defined policy guiding nurses on communication skills, service psychology and behavior. Participants frustratingly expressed limited attention from authorities in addressing workplace violence. Some cited the failure of concerned health authorities in recognizing workplace violence as a pressing issue. Others disappointedly stated authorities going to media outlets to castigate nurses which provoke public mistrust and hatred for nurses. *“Our bosses are not supportive; they did not regard violence as an important problem. They did not regard security as a problem”* said Jainaba, a female Registered Nurse Midwife from facility WII-1. Musa, a male Community Health Nurse at facility WI-7 said: “*I have seen instances where people go to the media, telling the population that nurses are bad, nurses are this, nurses are that, with all that stuff. And this helps to create enmity between the population and nurses”.*

## Discussion

This study represents the first to document the prevalence of workplace violence against nurses in the Gambia. Findings in 219 nurses indicate a rather high (62.1%) prevalence of exposure to workplace violence 12 months prior to the study. The most common form was verbal abuse. The primary perpetrators of reported incidents were patients’ escorts/relatives and patients themselves. The outpatient department was reported as the site where most violent incidents occurred. Qualitative feedbacks from participants attributed the perceived reasons of workplace violence mainly to attitude problems on the part of nurses and patients, understaffing and shortage of drugs, security vacuum and lack of management attention to workplace violence.

Regarding the prevalence of workplace violence, despite some variations in the definition of violence, targeted health professionals, sample size and methods used, the prevalence in this study (62.1%) is comparable to, but higher than several international studies [3, 4, 6, 14]. In general, nurses in the Gambia have higher rate of exposure (62.1%) to both verbal (59.8%), physical (17.4%) and sexual harassment (10%) than many country studies [15]. The higher prevalence of violence in our study could be explained by personal, societal and institutional factors.

Findings from interviews with participants indicate that miscommunication between providers (nurses) and care seekers (patients and their escorts) often culminate into wrangling. This has been attributed to the inability of some nurses to understand and respond appropriately to patients’ needs; a situation that often triggers aggressive reactions from patients and their escorts. The plausible explanation for such poor service behaviors could be lack of training as nearly 88% of participants indicated not receiving any form of training on violence recognition, prevention and management. As highlighted by Kamchuchat et al. 2008, training is an essential element of an effective violence prevention program. Their findings showed that training could reduce the risk of verbal abuse by 40% [6]. Similarly, a study on workplace violence against nurses in Texas in the USA revealed staff training/education/awareness as one of the most successful strategies for preventing workplace violence directed towards nurses [16]. Our study further revealed that nurses at times give clients the cause to abuse them by making offensive utterances to patients. Patients and escorts often come to health care facilities in stressful states; if nurses are not prepared to accommodate such emotions with restraint, it often results in aggressive confrontations between them and care seekers.

Similar to many of the previous studies, patients’ relatives/escorts and patients were reported as the main sources of violence. These results were comparable to previous researches in Ghana [17], Egypt [18], Palestine [5], Turkey [19], Taiwan [4], Thailand [6], Iran [7], India [20] and Iraq [21]. A study on workplace violence toward emergency department staff in Jordanian hospitals similarly reported patients and their relatives as the main source of violence [22]. Our study participants cited communication gap between clients and nurses, delay in services due to understaffing, shortage of drugs and supplies as the key reasons provoking aggressions from patients and their escorts/relatives. Some public preconceptions put Gambian nurses in an unfavorable situation. For instance, patients or their escorts construe nurses as ‘inefficient’ even if this is as a result of understaffing. Such preconception could unduly influence the behavior of patients and their escorts/relatives in negative ways toward nursing staff.

The reflection of participants suggests that nurses’ approach towards clients and work sometimes makes them victims of abuse. Such misconducts were reported to take the form of nurses uttering indecent languages towards patients, snubbing patient companies and reporting to work late. These behaviors among other possible reasons may have been affected by gaps in staff motivation. There was a joint national survey conducted in 2010 by the Ministry of Health & Social Welfare of the Gambia, Center for Innovation Against Malaria (CIAM) and West Africa Health Organization (WAHO) on the effects of incentives on health service providers’ performance in the Gambian public sector [23]*.* In this survey, nurses represent 88.3% of the participants. Sixty-five (65%) of respondents in this study expressed dissatisfaction with their living and working conditions including lack of access to equipment, drugs, utilities, sanitary facilities, mobility and opportunities for career advancement. The study indicates that these factors had negative effects on the motivation and performance of health care providers and were thus considered disincentives. It is possible that such disincentives were affecting the service behaviors of nurses towards clients and work as reported in our study.

The interview data suggest that inadequate drugs and supplies, long waiting time/delay in services often caused by understaffing were additional triggering factors of workplace violence. This finding is in accordance with the results of Kamchuchat and colleagues in Southern Thailand [6]. Reports from the interview in our study highlighted aggressive behaviors towards nurses from patients in instances of drug shortage. Similarly, staff shortage was described as an important triggering factor for reported incidents. Patients tend to express dissatisfaction in aggressive manners towards nursing staff when services get delayed due to long waiting times; a situation that often occurs where there is low staffing-patient ratio. These findings are in conformity with a 2016 study in Ghana that reported inadequate staff and infrastructure which lead to long waiting times [17]. Similar to our findings, this study indicates that the frustration that patients and their relatives may have to go through before they are attended to (due to long waiting times) and dissatisfaction with service could make them more inclined to abuse nurses verbally. A Jordanian study [22] similarly cited lack of resources, staff shortage and overcrowding as contributing factors to workplace violence in Jordanian hospitals. A study conducted in Egypt revealed comparable findings. This study cited increased workload and shortage of nursing staff as the main causes of violence perprated against nursing staff [18].

In the current study, majority of the events (59.5% of verbal abuse, 47.4% physical violence and 50% of sexual harassment incidents) occurred at the outpatient departments (OPD). This could be due to the sheer number of care seekers seen in the OPDs as compared to other units that exist in public secondary health care facilities in the Gambia. Besides, in the Gambia, the outpatient departments in public secondary health care facilities attend to accident and emergency victims in addition to regular outpatient care/services. Many studies recognized outpatient and emergency departments as particularly violent environments. These departments are usually attended by aggressive and stressed patients who are more likely to commit violence against health workers [5, 6]. Our results further indicate that a sizable majority of incidents (61.8% of verbal abuse and 68.4% of physical violence) occurred during the morning shift. This result could be explained due to high number of patient visits in the morning shift as compared to other shifts. This finding is congruent with Shogi’s et al. (2008) results in Iran reflecting the crowdedness of hospitals in different times of the day. In the present study, married nurses were more likely to report verbal abuse than respondents who were single. In the Gambia, married women are expected to take care of the domestic front in addition to their job demands, which may account for this difference. High domestic burden could result in nurses reporting to work late. As the qualitative results indicate, this might suggest the reason rendering late reporting to work by some nurses which often precipitates angry reaction from waiting patients. A qualitative study in Papua New Guinea indicates juggling family and work life as barriers to commit fully to patient needs and delays in coming to work [24]. Additional reason accounting for this difference could be that married nurses are generally more assertive. In contrast, a study in Lebanon reported that single nurses were more likely to have been exposed to verbal abuse as compared to married nurses [25].

The logistic regression analysis indicates that respondents with five (5) or less co-workers have higher odds of reporting verbal abuse than those with 11 or more co-workers. This could suggest that perpetrators were less inclined to direct verbal aggressions towards nurses where there are sheer number of staffs present. This is in agreement with a study in Northwest Ethiopia which indicates that the odds of workplace violence among nurses with 1–5 number of staff during the same working shift were 2 times higher as compared to those nurses who had more than 11 number of staff during the same working shift [26]. The plausible explanation for this is that when the number of nurses is low in a given shift, patient care could be delayed resulting in irritation from patients or their accompanying persons. Our results and the Ethiopian findings however, contradict an Egyptian study which indicates that about one third of nurses reported exposure to violence even in the presence of more than 10 other colleagues [3].

Our results indicate that nurses in Western I Health Region were more likely to report physical violence than their counterparts in Western II. Possible explanations accounting for this variation could be due to differences in location in terms of urban and rural setting for the health facilities in the two regions. All the public secondary health care facilities in Western I are situated in urban areas which are relatively more volatile than those in Western II where almost all the public secondary health care facilities are located in rural settings. Furthermore, there is higher patient burden in health facilities situtated in Western I.

### Limitations & strengths

This study has some limitations. One major limitation was the self-report design. This method depends on the ability of the participants to recall events in the last 12 months prior to the study, which is subject to recall bias. Moreover, due to the sensitive nature of the subject (sexual harassment in particular), the study results may have suffered reporting bias resulting to an underestimation of nurses’ exposure to workplace violence. This may have resulted to only 17.4% of physical violence and 10% sexual harassment reported in our study. Although the study was open to all categories of nurses, time and resource restrictions limited participation to only public secondary health care facilities in Western I and Western II Health Regions in the Gambia. Therefore, these results may not be generalized to the private and other categories of public health care facilities in the country. Due to time restriction for the data collection exercise, health care managers were not approached for interviews. This may have restricted information regarding management’s response to reported events and the reasons for their lack of actions as asserted by nurses. To this end, future research should take into account the concerns and limitations reported in this study. We would also recommend future studies to assess the outcomes/consequences of exposure to violence in order to attract urgent policy interventions to this occupational hazard.

Nonetheless, this study provides a preliminary investigation of the violence episodes against nurses in the Gambian context and may pave the way for further research. The data generated could also trigger policy response for the formulation of workplace violence prevention policy for Gambian nurses. Besides, limiting recall to the previous 12 months has been used successfully in other studies of violence at workplace [5, 27]. Efforts were made to maximise response rate through active follow-ups with participants. These efforts yielded an overall response rate of 98.25%. Therefore, the results could provide useful insight into the problem of workplace violence against nurses in the Gambia. Reporting bias as highlighted in other studies may also have been minimised. Emphasis on the benefits of the study findings in terms of heightening awareness and implementing effective policies on workplace violence were underscored to participants throughout. Besides, reporting bias that is due to involving head nurses in distributing and collecting completed questionnaires as done in some previous studies may have been minimized. The lead author personally distributed and collected all completed questionnaires from participants. The respondents were given assurance about confidentiality of their responses. This may have yielded objective reporting by participants about their workplace violence experiences. Additionally, the qualitative feedbacks from respondents allowed us to gain detailed and cross-verifying the study results (triangulation).

## Conclusions

This study revealed an overall prevalence of 62.1% to workplace violence suggesting that nurses in the Gambia are at high risk of workplace violence particularly verbal abuse. A great caution is thus needed because verbal abuse could cause serious psychological harm [28]. It could affect a nurse’s motivation and ability to offer effective care [29]. Most importantly, the persistent occurrence of verbal abuse could affect the attraction and retention of nurses within the health care system [30].

The most common perpetrators of violence were patients’ relatives/escorts and patients themselves. Qualitative feedbacks conclude understaffing, shortage of drugs & supplies, security vacuum and lack of management attention to workplace violence as the fundamental factors provoking violent incidents at work.

Given the said concerns, this study recommends that future interventions should be responsive to the need of the nursing staff for effective prevention of workplace violence. Thus, the Ministry of Health & Social Welfare of the Gambia should institute programs that will guide nurses to recognize, prevent and deal with workplace violence. A compulsory in-service education on workplace violence should be introduced for all nurses. The content of such programs should include communication skills, service psychology (understanding clients’ needs), service behaviour (how to respond appropriately to clients’ needs and personality improvement) and safety training (handling aggression and defusing hostile situations). It may also be necessary for nurse training institutions to include strategies to deal with assaults at workplace in their curricula, as incoming nurses may be exposed to this behavior.

There should be a public-based violence prevention education programs to heighten awareness on workplace violence. Mass education programs directed to improving the public image of nurses should be promulgated by the Health Promotion & Education Directorate of the Ministry of Health & Social Welfare of the Gambia. Such programs could give an accurate impression of nurses to the public. It could inform the public about the true value of nursing and how dispelling nursing stereotype could improve nurse-client relationship. In order to meet clients’ expectations, the government should ensure availability of adequate staff, drugs and supplies at all times. Staff protection by beefing up security at health facilities is fundamental. This should include assigning trained security guards, installing security cameras/video monitoring systems and check-in procedures for patient visitors.

## Additional file

Additional file 1: Workplace violence against nurses dataset. Dataset containing results of workplace violence assessment questionnaire for nurses in the Gambia. (XLS 197 kb)
